# Feasibility and potential effectiveness of an intensive trauma-focused treatment programme for families with PTSD and mild intellectual disability

**DOI:** 10.1080/20008198.2020.1777809

**Published:** 2020-07-14

**Authors:** Liesbeth Mevissen, Marjolein Ooms-Evers, Marike Serra, Ad de Jongh, Robert Didden

**Affiliations:** a Trajectum, Zwolle, The Netherlands; b Ambiq, Hoogeveen, The Netherlands; c Accare, Assen, The Netherlands; d Academic Centre for Dentistry Amsterdam (ACTA), University of Amsterdam and VU University Amsterdam, Amsterdam, The Netherlands; e Research Department, PSYTREC, Bilthoven, The Netherlands; f School of Health Sciences, Salford University, Manchester, UK; g Institute of Health and Society, University of Worcester, Worcester, UK; h School of Psychology, Queen’s University, Belfast, Northern Ireland; i Behavioural Science Institute (BSI), Radboud University, Nijmegen, The Netherlands

**Keywords:** PTSD, intensive EMDR, family trauma treatment, parental trauma, ACEs, mild intellectual disabilities, intergenerational transmission, autism, TEPT, EMDR intensivo, tratamiento trauma familiar, trauma parental, experiencias adversas infancia (ACEs), discapacidades intelectuales moderadas, transmisión intergeneracional, autismo, PTSD, 强化EMDR, 家庭创伤治疗, 父母创伤, ACE, 轻度智力障碍, 代际传递, 自闭症, • Multi problem families with mild intellectual disabilities or borderline intellectual functioning are at high risk for traumatisation and out of home placement of children.• KINGS-ID, a clinical trauma-focused family treatment programme, was found to be feasible and potentially effective.

## Abstract

**Background:**

Persons with mild intellectual disabilities or borderline intellectual functioning (MID-BIF; IQ 50–85) have a higher risk of being exposed to traumatic events and developing posttraumatic stress disorder (PTSD). EMDR therapy has shown to be applicable, safe and potentially effective for the treatment of PTSD in individuals with MID-BIF. However, in traumatized multi-problem families with MID-BIF and (impending) out of home placement of children, standard PTSD treatment in an outpatient setting may not be appropriate.

**Objective:**

To evaluate the feasibility and potential effectiveness of KINGS-ID, a six-week clinical trauma-focused treatment programme consisting of intensive EMDR therapy with parents and children, and parental skills training followed by two weeks of parent support at home.

**Method:**

Six families (nine parents of whom six had MID-BIF) and 10 children (all having MID-BIF) participated in the KINGS-ID programme. Seven parents and seven children had PTSD. Data were collected within a single case study design. For each family member data were collected during baseline (three measurements), treatment (seven weekly measurements), posttreatment (three measurements) and at follow-up (three measurements).

**Results:**

None of the family members dropped out. Within the first two treatment weeks all but one child and one parent no longer met PTSD symptom criteria. In both children and parents, trauma-related symptoms and daily life impairment significantly decreased following treatment and in parents a significant decrease in symptoms of general psychopathology and parental stress was found. Results were maintained at six-month follow-up.

**Conclusions:**

The findings of the current study are promising given that the treatment programme seems to offer new perspectives for traumatized multi-problem families with MID-BIF.

## Abstract (es)

**Antecedentes:** Las personas con discapacidades intelectuales moderadas o funcionamiento intelectual límite (MID-BIF; IQ 50-85) tienen un mayor riesgo de estar expuestas a eventos traumáticos y desarrollar trastorno de estrés postraumático (TEPT). La terapia EMDR o DRMO ha demostrado ser aplicable, segura y potencialmente efectiva para el tratamiento de TEPT en individuos con MID-BIF. Sin embargo, en familias traumatizadas con múltiples problemas y MID-BIF e inminente relocalización de niños fuera del hogar, el tratamiento con EMDR estándar en modalidad ambulatoria puede ser inapropiado.

**Objetivo:** Evaluar la factibilidad y potencial efectividad de KINGS-ID, un programa clínico de seis semanas de tratamiento centrado en el trauma, consistente en tratamiento EMDR intensivo con padres y niños, y un entrenamiento de habilidades parentales seguido por dos semanas de apoyo parental en el hogar.

**Método:** Seis familias (nueve padres, de los cuales seis tenían MID-BIF) y 10 niños (todos con MID-BIF) participaron en el programa KINGS-ID. Siete padres y siete niños tenían TEPT. Los datos fueron recopilados en un diseño de estudio de caso único. Para cada miembro de la familia los datos fueron obtenidos durante el inicio (tres mediciones), el tratamiento (siete mediciones semanales), post-tratamiento (tres mediciones) y seguimiento (tres mediciones).

**Resultados:** Ningún miembro de las familias desertó. Durante las dos primeras semanas de tratamiento, todos excepto un niño y un padre no cumplían criterios sintomáticos para TEPT. Tanto en niños como en los padres, los síntomas relacionados con el trauma y el deterioro en la vida diaria disminuyeron significativamente siguiendo el tratamiento y en los padres se encontró una disminución significativa en síntomas de psicopatología general y de estrés parental. Los resultados se mantuvieron a los seis meses de seguimiento.

**Conclusiones:** Los hallazgos de este estudio son promisorios, dado que el programa de tratamiento parece ofrecer nuevas perspectivas para familias traumatizadas con múltiples problemas y MID-BIF.

## Abstract (zh)

**背景:** 患有轻度智力障碍或具有边缘智力功能 (MID-BIF; IQ 50-85) 的人暴露于创伤事件并发展为创伤后应激障碍 (PTSD) 的风险更高。 EMDR治疗已被证明对于MID-BIF患者的PTSD治疗是适用, 安全并且可能有效的。但是, 在患有MID-BIF和 (即将) 离家安置的儿童的多问题创伤家庭中, 门诊环境中的标准PTSD治疗可能不合适。

**目的:** 评估KINGS-ID这项为期六周的聚焦创伤临床治疗方案的可行性和潜在有效性, 该方案包括与父母和孩子的强化EMDR治疗, 父母技能培训以及在家中接受父母两周的支持。

**方法:** 六个家庭 (9个父母中有6个患有MID-BIF) 和十个孩子 (都具有MID-BIF) 参加了KINGS-ID计划。七个父母和七个孩子患有PTSD。在单个案例研究设计中收集数据。对于每个家庭成员, 在基线 (3次测量), 治疗时 (每周7次测量), 治疗后 (3次测量) 和随访 (3次测量) 期间收集数据。

**结果:** 无家庭成员流失。在前两个治疗周内, 除一个孩子和一个父母之外, 其他参与者均不再符合PTSD症状标准。在儿童和父母中, 创伤相关症状和日常生活障碍在治疗后均显著降低, 在父母中发现了总体精神病态状态和父母压力的显著下降。结果在六个月的随访中维持。

**结论:** 鉴于该治疗方案似乎为遭受创伤的MID-BIF多问题家庭提供了新视角, 本研究结果很有前景。

## 1. Introduction

Children with mild intellectual disabilities or borderline intellectual functioning (MID-BIF, IQ 50–85 and adaptive functioning deficits in conceptual, social and practical domains) are at high risk of exposure to potentially traumatic events and the development of posttraumatic stress disorder (PTSD; Mevissen, Didden, & De Jongh, 2016a). PTSD is defined by exposure to actual or threatened death, serious injury or sexual violence resulting in intrusions, avoidance, negative alterations in cognitions and mood and marked alterations in arousal and reactivity (DSM-5; APA, 2013). PTSD negatively affects social, emotional, academic and physical development (Alisic, Jongmans, Van Wesel, & Kleber, 2011). Unfortunately, clinical experiences show that PTSD often is not recognized in persons with MID-BIF in that PTSD symptoms are wrongly attributed to MID-BIF, so called ‘diagnostic overshadowing’ (Jopp & Keys, 2001), or to other mental disorders considering the association between symptoms of PTSD and emotional and behavioural problems (Mevissen, Didden, & De Jongh, 2018; Mevissen, Didden, Korzilius, & De Jongh, 2016b).

Recent years have shown important developments concerning the diagnosis and treatment of PTSD in individuals with MID-BIF. For example, the DSM-5-based clinical interview DITS-ID (Diagnostic Interview Trauma and Stressors – Intellectual Disability; Mevissen et al., 2018; Mevissen, Didden, De Jongh, & Korzilius, 2020; Mevissen et al., 2016b) was developed to establish a PTSD diagnosis in children and adults with MID-BIF. Moreover, Eye Movement Desensitisation and Reprocessing (EMDR) therapy has been shown to be an applicable, safe, and potentially effective intervention for PTSD in children and adults with MID-BIF (Karatzias et al., 2019; Mevissen, Didden, Korzilius, & De Jongh, 2017).

Despite these promising developments, administering EMDR therapy may be difficult in traumatized children with MID-BIF living in multi-problem families, often with parents having MID-BIF as well. This especially applies to families with parents who have been exposed to traumatic events themselves, and who have children who are at risk of out of home placement. In clinical practice standard outpatient trauma treatment does not seem to be appropriate for these children for several reasons. First, parental trauma treatment is not incorporated in child trauma treatment. Parental trauma treatment might be of importance considering that parental PTSD symptoms can cause parenting limitations which can disrupt the development of the child (Van Ee, Kleber, & Jongmans, 2016). It is also suggested that trauma-related inadequate parenting skills may be accountable for an unsafe home environment marked by neglect and/or maltreatment (McGaw, Shaw, & Beckley, 2017). Further, parents need to be able to contribute to their child’s trauma recovery by giving emotional support, providing opportunities for positive experiences or by functioning as a co-therapist (Shapiro, Wesselmann, & Mevissen, 2017). It has been found that parental PTSD is associated with child distress and behavioural problems (Lambert, Holzer, & Hasbun, 2014). Thus, if parents are traumatized, participating adequately in the child’s treatment is likely to be difficult. This applies to parents of children without MID-BIF, but even more to those who have children with MID-BIF, given their greater dependency on caregivers. Second, standard outpatient trauma treatment may be inappropriate due to the accumulation of various other problems in traumatized multi-problem families. In clinical practice completing or even starting treatment is difficult due to for example financial problems, transport problems, lack of motivation and perseverance, planning problems and difficulties keeping appointments, physical health problems, emotional lability and lack of social support. Thus, to be effective, trauma-focussed interventions for children with MID-BIF, who live in multi-problem families with (a) traumatized parent(s), and who are at risk of out of home placement, should involve, trauma treatment of their parent(s) as well (Van Wesel, Boeije, Alisic, & Drost, 2012).

To overcome the shortcomings of standard outpatient trauma-focused treatment for this target group, the KINGS-ID (KINGS-Intellectual Disabilities) programme was developed. KINGS-ID is an intensive inpatient trauma-treatment programme for families in which one or more members have MID-BIF. The programme is an adapted version of KINGS (Wanders & Ploeg, 2017). KINGS (i.e. ‘kind in gezond systeem’) is a Dutch acronym for ‘child in healthy system’. The KINGS programme aims to contribute to a safe and healthy family system for children to grow up by enabling families to process traumatic memories and improve parenting skills. The original KINGS programme is theoretically well founded. Data on its effectiveness are not yet available.

The purpose of the present study was to evaluate the feasibility and potential effectiveness of KINGS-ID in six families. It was hypothesized that KINGS-ID would lead to (1) a significant decrease in PTSD symptoms and trauma-related daily life impairment in parent(s) as well as in children, (2) a significant increase in general mental health in parent(s), and (3) a significant decrease in parental stress. Our fourth hypothesis was that these treatment effects would be maintained at six-months follow-up.

## 2. Method

### 2. 1. Participants

Participants were six families (nine parents -aged 33 years and 9 months to 48 years and 5 months -, six mothers, one father and two stepfathers, and 10 children -aged 2 years and 9 months to 16 years and 9 months -, six boys and four girls). Two families participated with one child, and four families with two children. Three families were single parent families. Six parents and all ten children had MID-BIF. Five children had comorbid autism spectrum disorder (ASD). One parent without MID-BIF was diagnosed with dissociative disorder. Three parents with MID-BIF had comorbid disorders (ASD, ASD and substance use disorder, foetal alcohol syndrome and substance abuse disorder respectively). Seven parents and seven children fulfilled DSM-5 PTSD criteria at baseline. For each child and parent, adverse childhood experiences from the original ACEs (Adverse Childhood Experiences) framework were taken from the DITS-ID event section, using the definition of ACEs as used in the study of Vervoort-Schel et al. (2018) among out of home placed children with ID. ACEs as established by the children were emotional neglect (*n* = 10), having a parent with mental health problems (*n* = 10), having witnessed violence against a parent (*n* = 8), parental divorce (*n* = 8), sexual abuse (*n* = 5), physical neglect (*n* = 5), emotional abuse (*n* = 4), physical abuse (*n* = 3), parental substance abuse (*n* = 3) and parental incarnation (*n* = 1). Regarding the trauma history of the parents, childhood sexual abuse was reported by five of the six mothers. In five families a parent was victim of childhood physical abuse. Seven parents had a history of childhood emotional abuse and/or neglect. As a child, a majority of the parents had a parent with mental health problems and a majority experienced parental divorce. A minority of the parents reported childhood physical neglect and/or witnessing violence against a parent.

All families received outpatient home support for many years during which a variety of problems were addressed such as financial problems, housing, mental and physical health problems, addiction problems and delinquency. One parent received PTSD treatment as a child, another parent started outpatient trauma treatment, but dropped out for practical reasons. Two parents attempted suicide earlier. One of them was admitted to a psychiatric hospital several times. Another parent went through multiple treatments within addiction care. One parent was in a juvenile detention centre when she was a teenager. With regard to the participating children, one child received outpatient psychological treatment. None of the participating children received PTSD treatment before. Three of the participating children were outplaced in another treatment centre prior to the start of our study. In three families the mother had lost contact with one of her other children, not participating in the current study. In three families participating children were at risk for out of home placement. All mothers were divorced. None of the mothers had income from work.

### 2. 2. Design

Data were collected within a single case study design (Kazdin, 2011). For each family member data were collected during baseline (three measurements in a period of three to six weeks; the third baseline measurement was conducted at the first treatment day prior to the first EMDR therapy session), treatment (seven weekly measurements), posttreatment (three weekly measurements) and at follow-up at six weeks, three months and six months after treatment. All instruments were administered at each of these 16 assessment points.

This study was performed in accordance with the precepts and regulations for research as stated in the Declaration of Helsinki, and the Dutch Medical Research on Humans Act (WMO) concerning scientific research. The WMO was not applicable to the present study because (a) the questionnaires contained only a small number of items, (b) the study lacked random allocation, and (c) no ‘physical infringement of the physical and/or psychological integrity of the individual’ was to be expected.

### 2. 3. Inclusion and assessment

Within a period of one year, six families were referred to the treatment centre and each of them participated in the KINGS-ID programme. Inclusion criteria were: (1) at least one family member was diagnosed with MID-BIF, (2) parent(s) as well as children had been exposed to potentially traumatic events, (3) at least one child and one parent met DSM-5 PTSD criteria, and (4) despite earlier treatment children were at risk for out of home placement or out of home placement already had taken place and parents were strongly motivated to raise their child at home. All six families were eligible and participants (parents and children if aged 12 years or older) gave their written informed consent to participate in the study. They gave their permission to videotape all measurements, EMDR therapy sessions, parental skill training sessions and evaluation sessions. After a family had completed the six-week inpatient phase the next family started treatment.

Each family passed through a standardized assessment procedure. Together with the referring health care professionals the family visited the treatment centre where they received information about the treatment programme and the study. If the parents were interested to participate, the family was invited for an assessment meeting (first baseline measurement). During this meeting, each parent and – if possible – child set a series of personal treatment goals. Goals were for example: ‘I am able to manage my child in a calm way without shouting’, ‘I enjoy playing with my child’, ‘ I can talk to mama about fun things’ or ‘I am respectful to teachers’. Each goal was rated between 1 (goal not attained) and 10 (goal fully attained). Subsequently, a PTSD clinical interview was administered to each family member, and in parents general psychopathology and parental stress was measured. A home visit was part of the standard assessment procedure to provide details of the family living situation to adjust the intervention to the needs of the family. At least one week before the start of the treatment the second baseline measurement was taken. Assessment and preparation took six to eight weeks. This period was needed for the baseline measurements, but also for the home visit and several practical preparations such as a timely planning of EMDR sessions (in terms of therapist availability).

### 2. 4. Treatment

The treatment phase lasted eight weeks starting with a six-week inpatient programme. Intensive trauma treatment was offered by EMDR Europe certified therapists. Throughout the inpatient programme parents and children were assisted by family caregivers who had a background of cognitive-behavioural training and who were experienced in providing trauma-sensitive care. The latter was characterized by safety, opportunities for choice, collaboration, empowerment, attention for trauma triggers and understanding emotional and behavioural problems in the context of the person’s trauma history (Gardener, Iarocci, & Moretti, 2017). The caregivers were available the entire day and in case of emergency also during the night. After parental trauma-treatment the caregivers trained the parents in validating positive child behaviours by using video feed-back. Inpatient treatment was followed by two weeks of parent support at home (transfer).

### 2. 5. Setting

KINGS-ID was carried out at an inpatient unit located at a centre for child and adolescent psychiatry in the north-eastern part of the Netherlands, in close cooperation with a nearby organization specialized in the treatment and support of children with intellectual disabilities and their families. The families stayed in a separate family living unit. One family at a time was treated. Therapy sessions and family meetings were held in a central building. The family caregiver had an office adjacent to the family unit. The parents took care of their children and were responsible for cooking, shopping and related activities. Family caregivers invited parents and children to ask for help if needed, so the nature and scope of support varied and was tuned to the actual needs of the family members while keeping their personal goals in mind. Children were free from school. Parents and children participated in supervised leisure activities at the centre. Children returned to school immediately after discharge from inpatient treatment. The fathers who had a job organized permission to attend trauma treatment sessions and weekly evaluation meetings The latter took place in the presence of parents and children (if appropriate considering their mental age), an EMDR therapist, a family caregiver and the healthcare professional(s) who referred the family to KINGS-ID.

### 2. 6. EMDR therapy

The KINGS-ID programme started when the family arrived at the centre on a Sunday evening. At Monday morning the third baseline measurement was taken followed by the start of EMDR therapy with the parent. Where there were two parents the one with the most severe PTSD symptoms was offered EMDR therapy first. EMDR therapy of the child(ren) started immediately upon completion of parent treatment. Where there were two children the sequence was determined together with the parent(s). The first treatment measurement was taken after one week of intensive EMDR therapy.

For children as well as adults Eye Movement Desensitisation and Reprocessing (EMDR) therapy is a first-choice psychological treatment for PTSD (ISTSS, 2018). EMDR therapy is a protocolized, eight-phase psychotherapeutic approach, which aims to resolve symptoms resulting from disturbing and unprocessed life experiences (Shapiro, 2018).

In the present study history taking was performed by administering the DITS-ID (see Measurements). EMDR case formulation was standardized and based on the time line which was built during administration of the event section. A visualized overview of the events (‘ranking list’) was created in the sequence in which the memories of these events would be treated with EMDR. To that end each time line event was given a score between 0 and 10 (0 no distress and 10 highest distress) representing the subjective unit of distress (SUD) that was felt when the memory was kept in mind. Events were ranked from highest to lowest SUD score. Events with equal SUD scores were ranked from earliest to latest in life.

In the first treatment session EMDR was introduced. In the present study the Dutch protocol for children and adolescents (Beer & De Roos, 2017) was applied for children as well as parents. Instructions were given at a difficulty level which corresponded with the participant’s estimated mental age. A visual representation of a number scale was available to rate the SUD and VOC (Validity Of Cognition).

In the present study, EMDR therapy for parents and children comprised two 60 to 75 minute sessions a day during four days a week by multiple therapists. Between sessions family members were supported by the family caregiver who encouraged them to be physically active (Van Woudenberg et al., 2018). No subsequent treatment session was offered in case (a) none of the participant’s time line events elicited any disturbance when bringing up the corresponding memories, and (b) future templates (images of target behaviours, partially corresponding with personal goals) were successfully installed. In case a future template failed, the flashforward protocol was applied meaning that the most disturbing image of a feared disaster was desensitized (Logie & De Jongh, 2014).

### 2. 7. Main differences between KINGS-ID and the standard KINGS programme

There are three main differences between the KINGS-ID programme as described above and the standard KINGS programme.

1. The initial phase (one week) of the standard KINGS intervention was omitted for two reasons. Several studies suggested that a stabilization phase is not essential if traumatic memories are accessible (Lindauer, 2015). In addition, it was expected that (video) training to validate the child’s positive behaviours, which was carried out in the first week of the standard KINGS programme, would be more effective after EMDR treatment instead of before, knowing that (a) parental PTSD may nourish a negative view of the child and limit capacity to learn new skills (Alisic et al., 2011; Van Ee, Jongmans, Van der Aa, & Kleber, 2017) and (b) the presence of the family caregiver ensured that the child stayed in a safe environment where his or her basic needs were met. During the first week of the regular KINGS programme, caregivers also should provide the families with psychoeducation about the relationship between the traumatic events they had been exposed to and their emotional and behavioural problems. This should enhance treatment motivation. However, administration of the DITS-ID ensured that family members already were aware of this relationship since an in-depth survey of traumatic events related to daily life impairments was made. Therefore, extra time for psychoeducation was not considered necessary. Information about how EMDR therapy decreases trauma-related symptoms was given during (trauma) treatment.

2. For study design reasons a fixed time period instead of the flexible period for inpatient treatment was required. Clinical experience of previous cases showed that six weeks were on average sufficient to complete EMDR treatment of parents and children with MID-BIF, to practise parenting skills and to achieve personal goals.

3. Mainstream mental health interventions do not adequately meet the needs and characteristics of persons with MID-BIF, and therefore need to be adapted (Blankestein et al., 2019). Therefore, another difference with the standard KINGS programme was the addition of two weeks of daily parenting support at home to facilitate transfer of skills and maintenance of treatment results. Generalization was also fostered by a close cooperation between the family caregivers and the professional(s) who supported the family at home before the start of KINGS-ID and who would continue supporting the family after completion of the KINGS-ID programme. Finally, family caregivers and EMDR therapists were trained to adapt their level of communication to the cognitive deficits of children and parents. Techniques were used to motivate and continue treatment, communication was simplified by the use of easier language and visual cues, and the focus was limited to only one task at a time.

### 2. 8. Instruments

#### 2. 8. 1. PTSD diagnoses, number of PTSD symptoms and daily life impairment

The DITS-ID (Diagnostic Interview Trauma and Stressors – Intellectual Disability; Mevissen et al., 2018, 2020, 2016b) was used for diagnosing PTSD according to DSM-5 criteria. For children, the child version and the parent version were used. For parents, the adult version was used. The DITS-ID consisted of an event and a symptom section with answer categories ‘yes’, ‘no’ or ‘other’. Type A traumas as well as life events the child or adult had been exposed to were visualized on a timeline to help keep in mind the events when symptoms were asked for. Finally, a thermometer card was used to support the child or adult to indicate the interference score (0 = totally not, 8 = very much) representing the subjective level of daily life impairment. The child and parent version has good convergent validity and excellent interrater reliability (Mevissen et al., 2016b). Besides good convergent validity, the adult version of the DITS-ID has sufficient to excellent interrater reliability (Mevissen et al., 2020).

During the course of the study, exposure to new potentially traumatic events could take place, influencing PTSD symptom outcomes. Therefore, at each measurement, before administration of the symptom section of the interview, the parent or child was asked as to whether new potentially traumatic events had occurred.

The DITS-ID was administered by a trained trauma therapist or psychologist. The time line was used to conceptualize EMDR treatment. The DITS-ID was administered to all family members including those without MID-BIF.

### 2. 9. General mental health

The SCL-90 (Symptom Checklist; Arrindell & Ettema, 2005) is a self-report measure of psychological distress and symptoms of psychopathology. It consists of 90 statements about the presence of symptoms in the past week. Items are scored on a Likert scale ranging from 1 (not at all) to 5 (extremely). The SCL-90 includes eight subscales: Agoraphobia, Depression, Somatization, Insufficient thinking and acting, Distrust and interpersonal sensitivity, Hostility, Sleeping problems, and Other problems. The SCL-90 has satisfactory psychometric properties (Arrindell & Ettema, 2005).

### 2. 10. Parental stress

The OBVL (Opvoedingsbelasting-vragenlijst [Parental stress – questionnaire]; Vermulst, Kroes, De Meyer, Nguyen, & Veerman, 2015) is a Dutch self-report measure to index parental stress. It consists of 34 items and five subscales: parent-child relationship problems, problems with parenting, depressive mood, role limitation, and health complaints. Items are scored on a Likert scale ranging from 1 (does not apply) to 4 (applies completely). Reliability and validity of the OBVL are good (Vermulst et al., 2015).

Since the psychometric properties of the SCL-90 and OBVL self-report measures have not yet been investigated in individuals with MID-BIF both questionnaires were administered with the help of a trauma therapist or psychologist.

### 2. 11. Interrater reliability

For the child version, parent version and adult version of the symptom section of the DITS-ID 20% randomly chosen video-taped interviews, equally distributed across the different study phases, consisting of 35 (child version and parent version) or 39 (adult version) questions per interview, were independently scored by a trained second observer on a question-by-question basis (answer yes or no/other). Mean percentage of agreement (agreements divided by agreements and disagreements) was 97.9 for the child version, 99.2 for the parent version and 98.4 for the adult version indicating excellent interrater reliability of recording (Cicchetti, 1994).

### 2. 12. Statistical analyses

Outcomes for (1) trauma symptoms of parents and children (total number of PTSD symptoms), (2) daily life impairment (Interference score), (3) general psychopathology, and (4) experienced parenting stress were plotted on a graphical display. Individual data (152 graphs) were interpreted by visual inspection by the first author and a second independent rater, following the guidelines of Lane and Gast (2014) for assessing baseline trend. Combined effect sizes (*Tau-U*) for baseline versus treatment, baseline versus post-treatment and baseline versus follow-up were calculated (Parker, Vannest, & Davis, 2011; Parker, Vannest, Davis, & Sauber, 2010; Vannest & Ninci, 2015). In case of improvement during baseline, a correction for baseline trend was administered. In the current study *Tau-U* could vary between −1 (maximum effect) and 0 (no effect). Level of significance was set at 0.001. Whether or not participants met the DSM-5 criteria for PTSD was assessed at each measurement point.

## 3. Results

### 3. 1. Course of parents’ symptoms

For parents, results show a significant decrease in trauma symptoms, trauma-related daily life impairment, general psychopathology as well as experienced parenting load (see Table 1). Results were maintained at posttreatment and at follow-up. The combined TAU’s indicate large to very large effect sizes.

**Table 1.** Combined values of TAU across parents.

	Baseline- treatment (*n*=9)		Baseline-post-treatment (*n*=7/*n*=6)^1^		Baseline-follow-up (*n*=7)2	
Measure	*Tau-U*	*CI 90%*	*Tau-U*	*CI 90%*	*Tau-U*	*CI 90%*
Number of trauma symptoms parents	−0.92*	−1<>-0.69	−0.90*	−1<>-0.59	−0.90*	−1<>-0.59
Daily life impairment parents	−0.91*	−1<>-0.68	−0.86*	−1<>-0.54	−0.83*	−1<>-0.51
General psychopathology parents	−0.88*	−1<>-0.65	−0.78*	−1<>-0.46	−0.83*	−1<>-0.51
Experienced parenting load	−0.91*	−1<>-0.68	−0.78*	−1<>-0.44	−0.87*	−1<>-0.56

*Significant at *p* < 0.001.

^1^For two parents one respectively two posttreatment measurements could not be taken. For ‘Experienced parenting load’ *n*=6 (for one parent one posttreatment measurement was missing).

2 For two parents two respectively three follow-up measurements could not be taken.

Mean number of trauma symptoms for parents (range 0–35) were 19.4 (*SD *= 8.1) at first baseline measurement, 10.2 (*SD *= 6.3) at first treatment measurement, 4.1 (*SD *= 4.7) at first post-treatment measurement, and 3.2 (*SD *= 2.5) at six months follow-up measurement. For trauma-related daily life impairment mean interference scores (range 0–8) were 7.0 (*SD *= 1.5), 3.4 (*SD *= 1.9), 1.1 (*SD *= 1.4), and 1.6 (*SD *= 1.3), respectively. For general psychopathology mean SCL-90 scores (clinical range >125) were 194.8 (*SD *= 37.5), 131.6 (*SD *= 24.7), 104.5 (*SD *= 24.7), and 106.0 (*SD *= 10.7) respectively. For experienced parenting load mean OBVL scores (subclinical range: scores 60–63; clinical range: >63) were 70.1 (*SD *= 7.0), 57.6 (*SD *= 12.8), 47.0 (*SD *= 14.6) and 44,0 (*SD *= 6.2), respectively.

### 3. 2. Course of symptoms of children

Table 2 shows that trauma symptoms and daily life impairment in children significantly decreased during treatment. Results were maintained at posttreatment and at follow-up with large effect-sizes.

**Table 2.** Combined values of TAU across children.

	Baseline-treatment (*n*=6)^1^		Baseline-post-treatment (*n*=5)^2^		Baseline-follow-up (*n*=6)	
Measure	*Tau-U*	*CI 90%*	*Tau-U*	*CI 90%*	*Tau-U*	*CI 90%*
Number of trauma symptoms children	−0.68**	−0.96<>-0.40	−0.60*	−0.98<>-0.23	−0.80	−1<>-0.45
Daily life impairment children	−0.74**	−1<>-0.46	−0.73*	−1<>-0.36	−0.74	−1<>-0.40

**Significant at *p* < 0.001. *Significant at *p* < 0.01.

^1^Considering mental age, for four children the child version of the *DITS-ID* could not or only partially been administered.

2 For one child two posttreatment measurements could not be taken.

Mean number of trauma symptoms for children (range 0–35) were 12.2 (*SD *= 9.4) at first baseline measurement, 8.5 (*SD *= 8.7) at first treatment measurement, 3.3 (*SD *= 4.8) at first post-treatment measurement, and 2.0 (*SD *= 2.7) at six month follow-up. Mean scores for trauma-related daily life impairment (range 0–8) were 5.0 (*SD *= 2.8), 3.0 (*SD *= 3.1), 0.7 (*SD *= 1.5)and 1.3 (*SD *= 2.0), respectively.

### 3. 3. Course of symptoms of children as reported by parent(s)

Table 3 displays the course of trauma-related problems in children as reported by their parent(s). Results show a significant decrease in trauma symptoms and trauma-related daily life impairment during treatment which remained at posttreatment and follow-up with large to very large effect sizes.

**Table 3.** Combined values of TAU across children as reported by the parent.

	Baseline-treatment (*n*=10)		Baseline-post-treatment (*n*=8)^1^		Baseline-follow-up (*n*=9)^2^	
Measure	*Tau-U*	*CI* 90%	*Tau-U*	*CI* 90%	*Tau-U*	*CI* 90%
Number of trauma symptoms children	−0.69*	−0.90<>-0.47	−0.79*	−1<>-0.50	−0.81*	−1<>-0.53
Daily life impairment children	−0.62*	−0.84<>-0.41	−0.93*	−1<>-0.63	−0.82*	−1<>-0.52

*p < 0.001.

^1^For two children two posttreatment measurements could not be taken.

2 For one child two follow-up measurements could not be taken.

Mean number of trauma symptoms for children as reported by their parents were 12.9 (*SD *= 7.6) at first baseline, 10.1 (*SD *= 8.7) at first treatment, 2.9 (*SD *= 2.3) at first post-treatment, and 2.5 (*SD *= 2.4) at six month follow-up. For child trauma-related daily life impairment according to the parents, these scores were 5.4 (*SD *= 2.2), 5.1 (*SD *= 2.3), 0.8 (*SD *= 0.9), and 1.2 (*SD *= 1.2), respectively.

### 3. 4. Course of PTSD in parents

At the first baseline measurement, seven of the nine parents fulfilled DSM-5 PTSD criteria. Four parents no longer met DSM-5 PTSD symptom criteria in the first treatment week. After two weeks all except one parent no longer met PTSD criteria. The number of EMDR therapy sessions varied from three (i.e. for the parent who did not meet PTSD criteria at the first baseline measurement) to 27 for the parent with PTSD and a dissociative disorder. Results were maintained, except for one parent who showed a temporary relapse. At six-month follow-up none of the parents fulfilled the criteria for PTSD.

### 3. 5. Course of PTSD in children

According to the child version of the DITS-ID, two of the six children met full PTSD criteria at the first baseline measurement. Within their first EMDR treatment week they no longer met PTSD criteria and results were maintained at six month follow-up. According to their parents, as measured with the parent version of the DITS-ID, seven of the 10 children met PTSD criteria at the first baseline measurement. Two of them no longer met PTSD criteria within their first EMDR treatment week. Both other children lost their PTSD diagnosis after PTSD remission of the parent just before the start of their own EMDR treatment. Two children temporary relapsed. The number of EMDR sessions varied from two to 16. None of the children fulfilled the PTSD criteria at six month follow-up.

### 3. 6. Trauma memories

Except for one parent (none of the negative events at the DITS-ID time-line elicited any disturbance; that is, SUD-scores were 0) all participants underwent EMDR therapy to resolve their disturbing memories. Across family members a great variety of trauma histories and negative life events could be detected. Many of them met ACEs criteria. Without exception, distressing memories related to sexual abuse, physical abuse/domestic violence and emotional abuse needed to be targeted with EMDR. During the course of treatment, memories with lower initial levels of distress became neutral without having to be targeted with EMDR.

## 4. Discussion

As far as we know, this is the first study to investigate the feasibility and potential effectiveness of an intensive trauma focussed treatment in families with MID-BIF. The programme provided intensive, inpatient trauma treatment (EMDR therapy) for both parents and children, that was imbedded in a trauma-sensitive environment. Subsequently, the programme focussed on improving parenting skills. None of the nineteen family members dropped out. In both children and parents trauma-related symptoms and daily life impairment significantly decreased and in parents a significant decrease of symptoms of general psychopathology and experienced parenting stress was found. Results were maintained at six-months follow-up. Therefore, the present results were in support of our hypotheses.

It should be noted that despite these promising results, in one of the families out of home placement of the (adolescent) child could not be prevented, despite intensive trauma treatment and educational support. This child and one of its parents had MID-BIF and comorbid ASD whereas the other parent had MID-BIF and other severe comorbid disorders. Probably such combination of severe difficulties in one family impede good enough parenting. Further, in two other family members with MID-BIF and autism spectrum disorder a temporary relapse occurred due to a new stressor. Addressing these problems by autism focussed educational support turned out to be helpful. The aforementioned findings suggest that for persons with MID-BIF and comorbid ASD the programme is effective in dealing with past trauma and stressors, but not in preventing future trauma and stressor related problems. This might be a subject of future research considering that until now few studies investigated trauma in persons with ASD (Lobregt-van Buuren, Sizoo, Mevissen, & de Jongh, 2019). Finally, one of the participating younger children with MID-BIF relapsed after exposure to a new traumatic event. This child unintentionally overheard his parents talking and drew the wrong conclusion to have to go back to live with the person who had abused him seriously and for a long time. After one session of EMDR therapy in presence of the parents PTSD symptoms disappeared.

The present study might be of importance given that families with MID-BIF are vulnerable. For example, analysis of national registers in Norway revealed that parental custody was revoked for 30–50% of children with parents with MID-BIF and that parents with MID-BIF accounted for 20–25% of all custody cases (Tøssebro, Midjo, Paulsen, & Berg, 2017). Also in the USA parents with MID-BIF have been found to be generally over-represented in Child Protection Services (CPS), often having a history of being abused or neglected themselves in childhood (LaLiberte, Piescher, Mickelson, & Lee, 2017). In the UK, McGaw et al. (2017) identified a tentative link between mental disorders and emotional abuse for children of parents with MID-BIF when childhood trauma and psychopathology in parents coexists. These findings are in line with studies among parents without MID-BIF, suggesting that parents, who have been abused (physically, sexually, emotionally) or neglected themselves during their own childhood, have an enhanced risk for maltreatment of their offspring by showing less warmth, a lack of involvement and more verbal or physical aggression (Assink et al., 2018; Berlin, Appleyard, & Dodge, 2011). The transgenerational aspect of trauma has also been exhibited by a recent analysis of case-files of children referred to a Dutch national centre for residential youth care for children with MID-BIF (Vervoort-Schel et al., 2018). It was found that almost half (49.3%) of the 69 children experienced at least 2 ACEs from the original ACEs framework and that the number of ACEs in children was related to the presence of ACEs in parents.

In the present study, for all except one child out of home placement could be prevented. This finding is in line with findings from studies that show that limitations in intellectual ability itself do not equate with poor parenting abilities (LaLiberte et al., 2017).

The present study has several limitations and strengths. Clearly, to determine the efficacy of interventions randomized controlled trials are pivotal. On the other hand, complex clinical treatment programmes are challenging in that such study designs need larger samples, randomization, and active control conditions. These requirements are often unattainable in clinical practice. Despite the limited number of six families, the *n* = 1 design which included multiple assessments before, during and after treatment, is a first step in providing evidence for positive effects of a combined parent and child trauma focussed treatment. Further, the current study lacked a random allocation to baseline length. However, the treatments were spread over a one-year period and the baseline length varied between three and six weeks. Another limitation of the present study is that it focussed on the feasibility and effectiveness of the trauma treatment (EMDR therapy) whereas the KINGS-ID programme includes other interventions, such as a trauma-sensitive environment and parenting skills training performed by the family caregivers. For future studies it is recommended to investigate the effectiveness of the KINGS-ID programme, with a more detailed focus on the interventions of the family caregivers to unravel the contribution of different treatment components to positive changes in aspects of parenting behaviours and child development. Finally, it is a limitation of the study that not all assessors who took the measurements were independent.

One strength of the present study is the use of a reliable and valid clinical interview; that is, the DITS-ID. This assessment procedure provided clinicians with a standardised and replicable procedure to conceptualise EMDR treatment in adults and children with MID-BIF and a history of multiple ACEs and other stressful life events. An additional strength of the current study is that the frequency of measurements allowed for a more detailed analysis of the study outcomes which affords additional, relevant information for clinical practice. For example, in almost all parents and children PTSD was in remission within two weeks of in-patient trauma treatment. This is in line with recent research findings from intensive trauma-focused interventions for individuals without MBID-BIF and severe PTSD, showing a significant decline in PTSD symptom severity in a short period of time with low drop out (Hendriks et al., 2017; Van Woudenberg et al., 2018). Most children already had lost their PTSD diagnosis before the start of their own EMDR therapy probably as a result of improved functioning of their parent(s). Whether traumatisation of children might partly be caused by trauma-related parenting behaviour, meaning that parental trauma-focused treatment should be part of therapy programmes for traumatized youth, needs further exploration in future research. Finally, future research could address the possibility of shortening the current treatment programme considering the very fast PTSD symptom decrease and the differences in the size of the families.

In conclusion, the findings of the current study are promising given that the KINGS-ID treatment programme seems to offer new perspectives for families with MID-BIF and chronic, pervasive, intergenerationally transmitted problems which are considered difficult to resolve and go along with long term personal suffering and high costs of psychological and medical care and out of home placement of children.
